# Autoimmune Polyglandular Syndrome Type 3 and Overlapping Autoimmune Endocrinopathies: A Case Report

**DOI:** 10.7759/cureus.88574

**Published:** 2025-07-23

**Authors:** Divyashri R Nagarajan, Daya Mani Jacob, Niyas Khalid Ottu Para

**Affiliations:** 1 Internal Medicine, Burjeel Medical City, Abu Dhabi, ARE

**Keywords:** autoimmune polyglandular syndrome type 3, autoimmune thyroiditis, endocrine dysfunction, levothyroxine resistance, multiple autoimmune syndrome

## Abstract

Autoimmune polyglandular syndrome type 3 (APS type 3) is defined by the coexistence of autoimmune thyroid disease with other autoimmune disorders, excluding adrenal insufficiency. It is a common but often under-recognized entity, particularly in young women with multiple autoimmune conditions.

We report the case of a 27-year-old woman with a longstanding history of type 1 diabetes mellitus (T1DM) who presented with multiple episodes of diabetic ketoacidosis to the emergency department. She presented with fatigue and had poor glycemic control (HbA1c 11.3%). She was further diagnosed in the ward with refractory autoimmune hypothyroidism, pernicious anemia, celiac disease with poor dietary adherence, and proliferative diabetic retinopathy requiring retinal intervention. Physical examinations, blood workup, and radiological investigations revealed no signs of adrenal insufficiency or pituitary adenoma. The combination of autoimmune thyroid disease, T1DM, pernicious anemia, celiac disease, and systemic autoimmunity, in the absence of adrenal involvement, is consistent with a diagnosis of APS type 3 with overlapping subtypes.

This case illustrates the clinical complexity and diagnostic challenges of APS type 3. Early identification and multidisciplinary management of coexisting autoimmune disorders are essential for improving long-term outcomes and reducing complications. Attention to dietary adherence, appropriate thyroid hormone dosing, and ongoing screening for evolving autoimmune involvement are critical elements in the care of such patients.

## Introduction

Autoimmune polyglandular syndrome (APS) encompasses a spectrum of rare, heterogeneous disorders characterized by autoimmune-mediated dysfunction of multiple endocrine glands. These disorders may also involve non-endocrine autoimmune manifestations, making their diagnosis complex and their clinical presentation highly variable. APS is classified into distinct subtypes, most notably APS type 1, APS type 2, and APS type 3, based on the specific combination of affected organs, age of onset, and genetic predisposition [1].

APS type 1 is a rare childhood-onset condition caused by mutations in the AIRE gene. It typically presents with a triad of chronic mucocutaneous candidiasis, hypoparathyroidism, and adrenal insufficiency [2]. APS type 2, also known as Schmidt's syndrome, is polygenic, more prevalent, and primarily affects adults. It involves autoimmune adrenalitis, autoimmune thyroid disease (either Hashimoto’s thyroiditis or Graves' disease), and type 1 diabetes mellitus (T1DM) [3].

APS type 3 is defined by the presence of autoimmune thyroid disease (such as Hashimoto’s thyroiditis or Graves’ disease) in association with other autoimmune conditions, without adrenal involvement. These may include type 1 diabetes, vitiligo, pernicious anemia, or celiac disease. APS type 3 is more common than the other subtypes, with an estimated incidence of 1 in 20,000 live births and a female predominance (male-to-female ratio of approximately 1:3) [4].

Understanding APS is critical for clinicians, particularly those managing patients with type 1 diabetes or autoimmune thyroid disease, as additional autoimmune conditions may emerge over time. Delayed diagnosis can result in life-threatening complications such as unrecognized adrenal insufficiency. Moreover, the coexistence of non-endocrine autoimmune diseases, such as vitiligo, pernicious anemia, or celiac disease, further complicates timely recognition and treatment [5].

Given its rarity and diverse presentations, APS, especially type 3, is often underrecognized in general medical practice. Case reports play a vital role in expanding clinical awareness and highlighting unusual combinations of autoimmune conditions.

Here, we present a unique case of APS type 3 with an uncommon combination of endocrine and non-endocrine features, contributing to the growing literature on this complex and evolving syndrome.

## Case presentation

A 27-year-old woman with a longstanding history of T1DM, diagnosed at the age of one, presented to the emergency department with abdominal pain, nausea, vomiting, and fatigue. She was diagnosed with diabetic ketoacidosis (DKA) and admitted to the ICU, where a standard DKA protocol was initiated, after which she was transferred to the inpatient ward.

Despite metabolic stabilization, the patient’s clinical trajectory remained atypical. She continued to report persistent fatigue and was noted to have frequent episodes of hypoglycemia with impaired awareness, despite adherence to a basal-bolus insulin regimen (aspart 10 units TID and glargine 1 unit daily). Her chronic glycemic control was poor, with a recent hemoglobin A1c of 11.3%. This raised concern for underlying endocrine dysregulation beyond her known diabetes.

On examination, she appeared thin but well-nourished. There were no overt signs of thyroid enlargement or orbitopathy. Neurological examination was unremarkable, with preserved sensation and intact distal pulses, ruling out peripheral neuropathy or vascular compromise.

**Figure 1:**
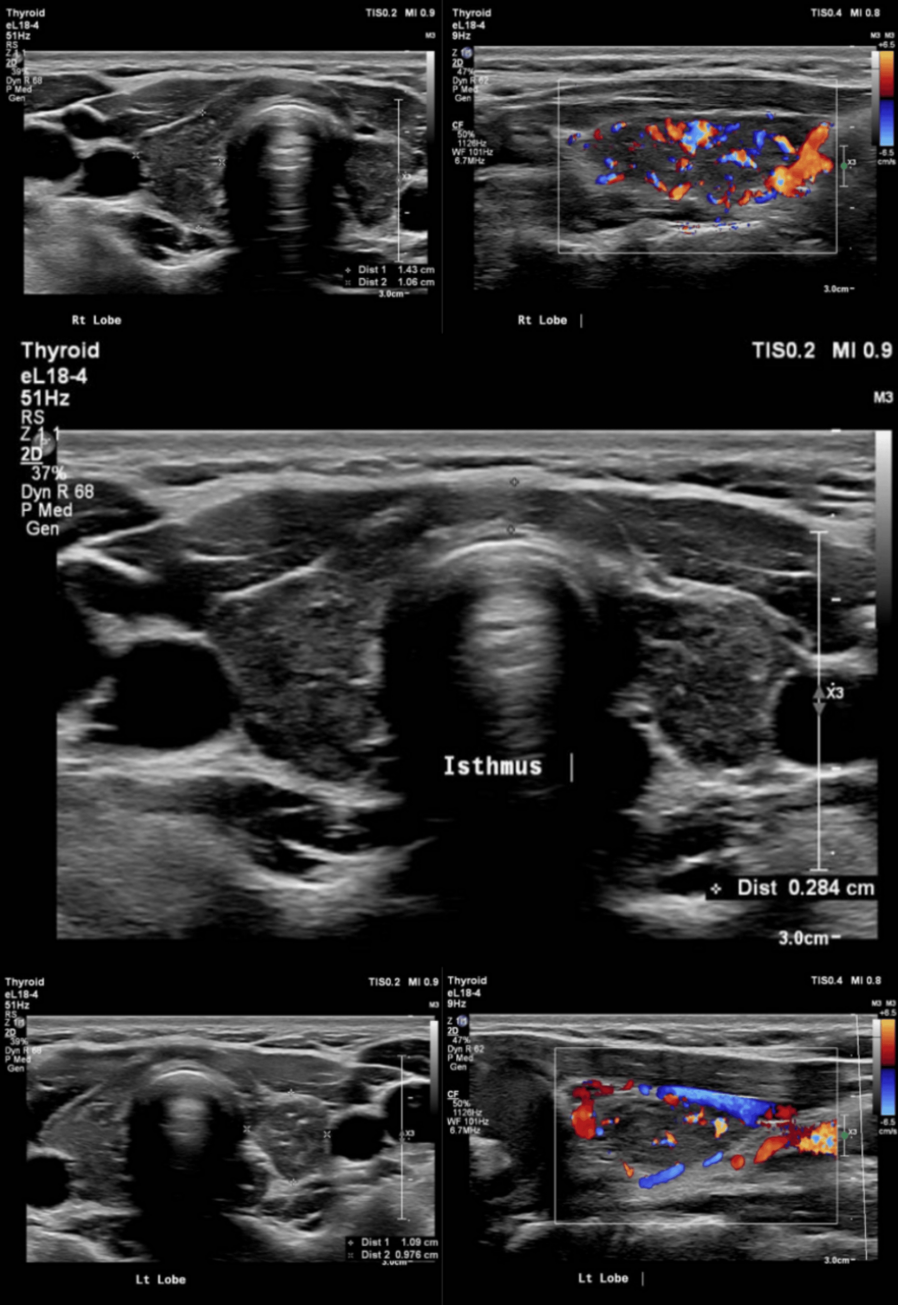
Ultrasound of the thyroid showing lobes and isthmus appearing normal in size having heterogeneous echotexture. Diffuse increased vascularity is also seen, suggestive of thyroiditis

Given the atypical course and systemic symptoms, a comprehensive endocrine and autoimmune workup was initiated. Thyroid function tests revealed elevated thyroid-stimulating hormone (TSH) with low T3 and low-normal free T4, serology was positive for anti-TPO and anti-TSHR antibodies, and ultrasound revealed features suggestive of thyroiditis (Figure 1), confirming autoimmune thyroid disease. She was on levothyroxine 100 mcg/day, but persistent biochemical hypothyroidism raised suspicion for an underlying central process.

**Figure 2:**
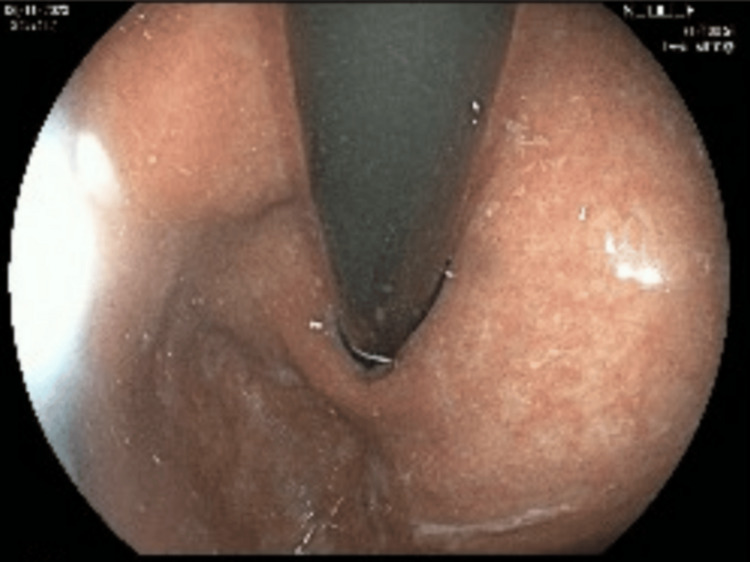
Scalloping of duodenal mucosa suggestive of ongoing celiac disease

Further investigations uncovered additional autoimmune associations. She tested positive for gliadin IgA/IgG, and duodenal biopsy confirmed villous atrophy (Figure 2), diagnostic of celiac disease, though she admitted to non-adherence to a gluten-free diet. Her history was also notable for vitamin B12 deficiency, with positive intrinsic factor and parietal cell antibodies, suggestive of autoimmune gastritis/pernicious anemia. Additionally, the antinuclear antibody (ANA) test was positive, suggesting possible systemic autoimmune activity. However, no further evaluation was pursued in this context.

**Figure 3:**
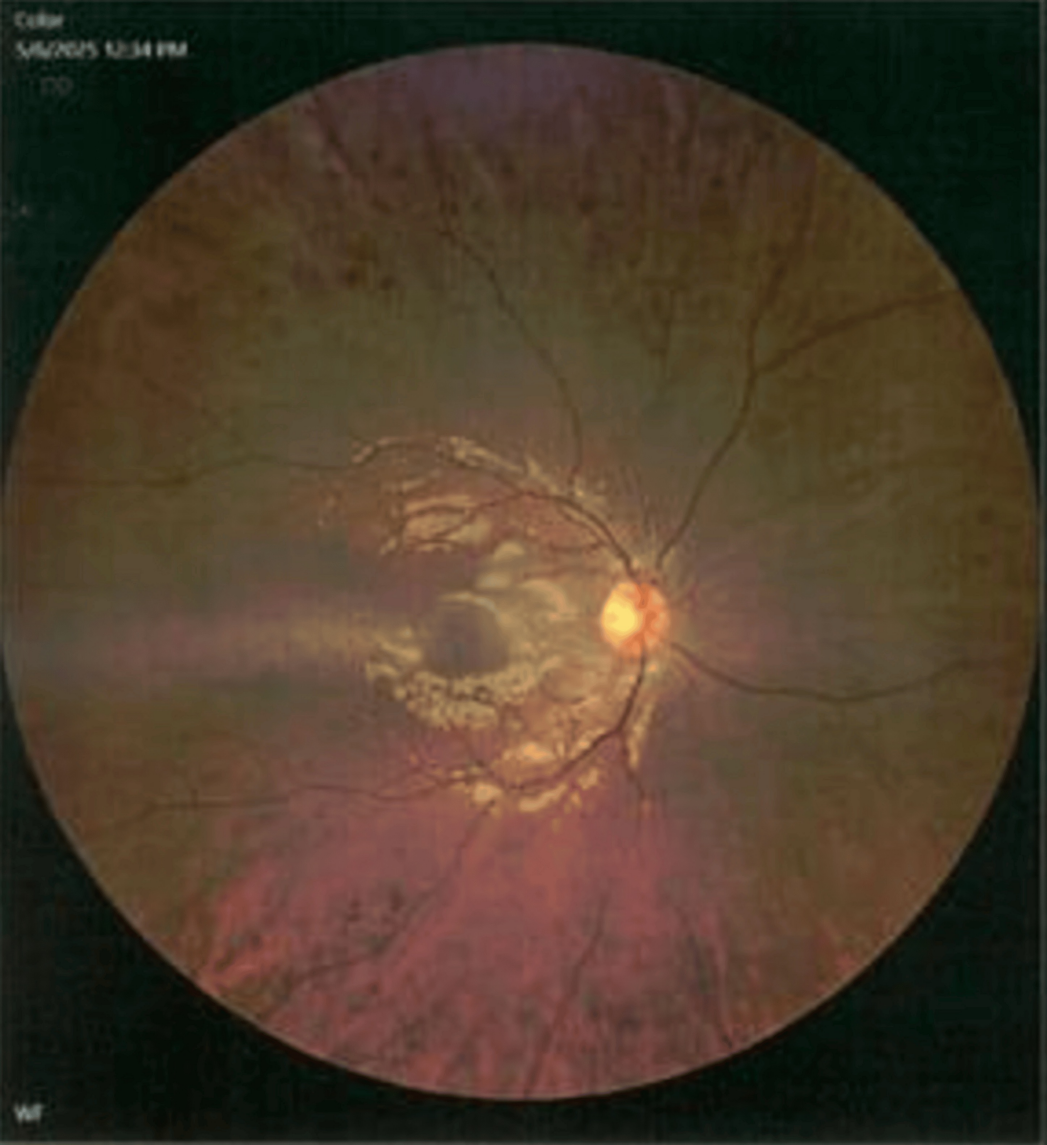
Fundoscopy showing proliferative diabetic retinopathy (right eye)

Her medical history was also significant for hyperlipidemia on rosuvastatin-ezetimibe and proliferative diabetic retinopathy (Figure 3) for which she was scheduled for intravitreal Vabysmo injections.

During her hospital stay, fatigue and endocrine symptoms persisted despite the correction of DKA. Given the possibility of adrenal involvement, additional hormonal assessments were performed. Her ACTH and IGF-1 levels were low, as was her T3. However, morning cortisol was normal, and a Synacthen stimulation test demonstrated adequate adrenal reserve, effectively ruling out primary adrenal insufficiency. Key laboratory findings are summarized in Table 1.

**Table 1:** Key laboratory findings

System	Test	Result	Reference Range	Interpretation
Pancreas	Hemoglobin A1c	11.30%	4-5.7%	Poor glycemic control → suggests Type 1 Diabetes Mellitus (APS 3A)
Thyroid	TSH	29.70 mIU/L	0.27-4.2 mIU/L	Elevated
	Free T4	11.4 pmol/L	12.3-20.3 pmol/L	Low-normal
	Total T3	0.92 nmol/L	3.10-6.80 nmol/L	Low
	Anti-TPO Antibody	234 IU/mL	<34 IU/mL	Strongly positive
	Anti-TSHR Antibody	Positive	<1.5 IU/L	Confirms autoimmune thyroid disease (required for APS Type 3)
Gasterointestinal	Gliadin IgA / IgG	Positive		Suggests celiac disease (APS 3C)
	Duodenal Biopsy	Villous scalloping		Supportive of active celiac disease
	Vitamin B12	184 pg/mL	211 - 946 pg/mL	Deficient
	Intrinsic Factor IgG	12.9	<6 U/mL	Positive
	Parietal Cell Antibody	Strongly positive		Confirms autoimmune gastritis/pernicious anemia (APS 3B)
Rheumatology	ANA (Antinuclear Antibody)	Positive		Suggests systemic autoimmunity (APS 3D)
Adrenal	ACTH	Low (0.33–1.08 pmol/L)	1.6-13.9 pmol/L	Low
	Morning Cortisol	354 nmol/L	138-690 nmol/L	Normal
	Synacthen Test	Adequate response		Adrenal function intact → rules out Addison’s disease (not APS Type 2)
Other	IGF-1	14.2 nmol/L	10.7-37.1 nmol/L	Low-normal

**Figure 4:**
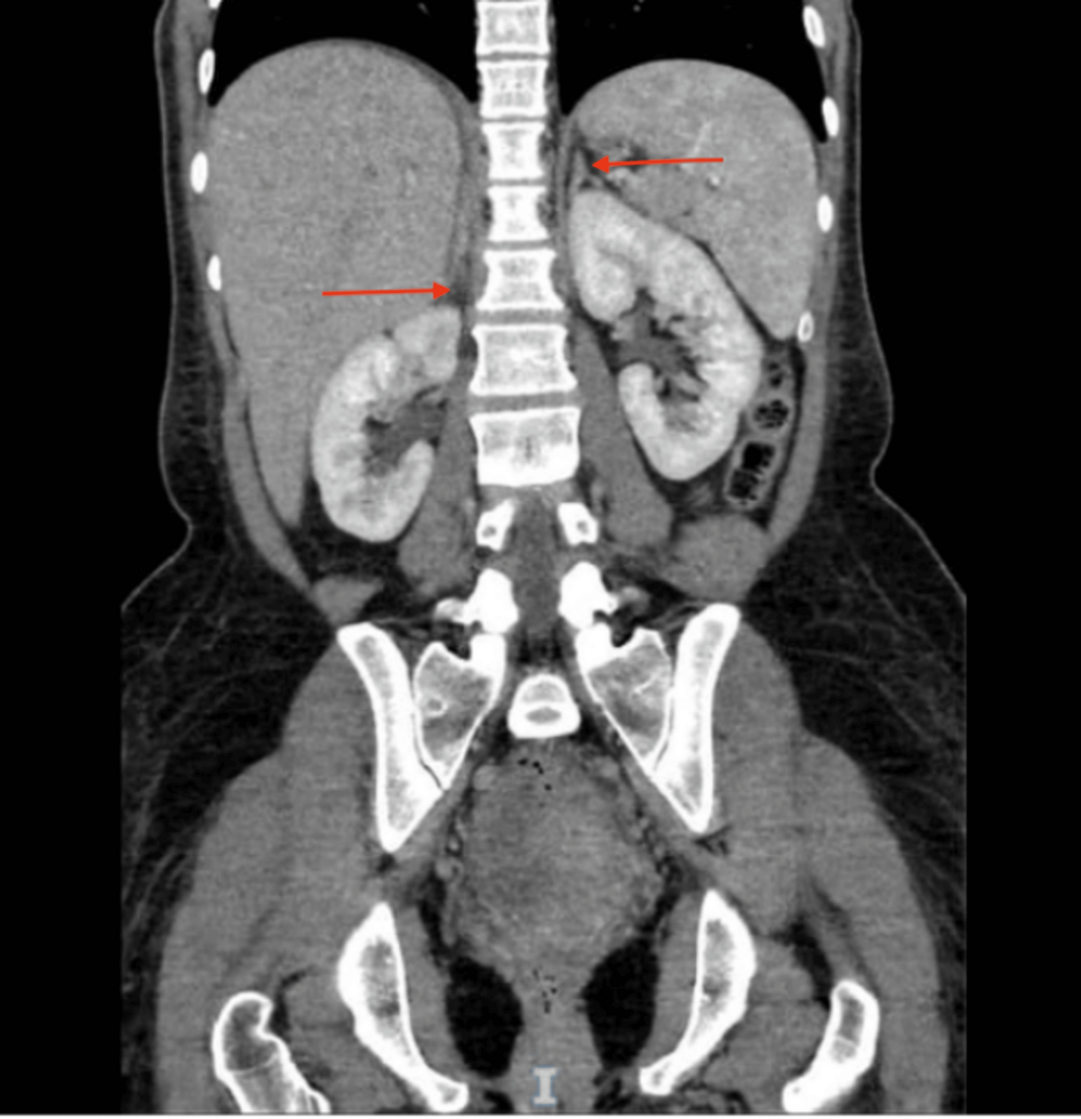
CT abdomen with contrast showing normal adrenal glands

**Figure 5:**
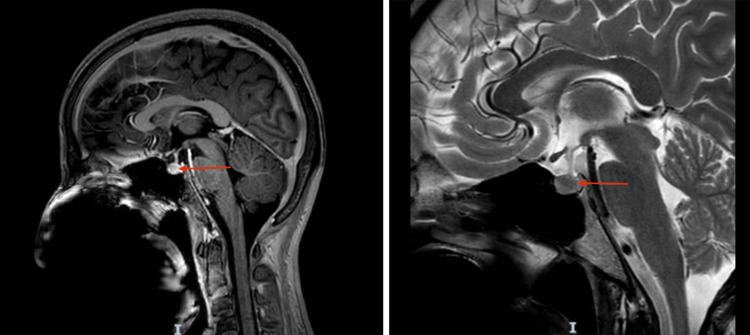
Sagittal section of MRI brain showing a normal pituitary gland

Imaging showed normal adrenal glands on CT (Figure 4) and an unremarkable pituitary MRI (Figure 5), excluding structural causes of central hypopituitarism.

Based on the combination of autoimmune thyroiditis, T1DM, celiac disease, pernicious anemia, and systemic autoimmunity, the patient met the diagnostic criteria for APS type 3. She was managed with adjustments to her levothyroxine dosage, optimization of insulin therapy, supportive management of her nutritional deficiencies, and guidance on adherence to a gluten-free diet. Her condition improved during hospitalization, and she was discharged in a stable state with plans for continued outpatient follow-up with endocrinology and pituitary specialists.

## Discussion

APS refers to a group of syndromes characterized by the immune-mediated dysfunction of multiple endocrine glands, often accompanied by autoimmune non-endocrine disorders. APS type 3, in particular, is subdivided into four subgroups, based on the association of one thyroid autoimmune disease with one or more of the other autoimmune conditions (Table 2).

**Table 2:** Classification of autoimmune polyglandular syndrome type 3 Source: [4]

APS Subtype	Criteria	
Type 3A	Autoimmune thyroiditis + Type 1 DM	
Type 3B	Autoimmune thyroiditis + Pernicious anemia	
Type 3C	Autoimmune thyroiditis + Celiac disease	
Type 3D	Autoimmune thyroiditis + Positive ANA (systemic autoimmunity)	

Our patient presents with a constellation of five distinct autoimmune conditions, fulfilling the diagnostic criteria for APS type 3 with overlapping subtypes. She has autoimmune thyroiditis, confirmed by elevated TSH and positive anti-TPO and TSH receptor antibodies, which is the central feature of APS type 3. Poor glycemic control with an HbA1c of 11.3% and a history of T1DM, consistent with APS type 3A. The presence of vitamin B12 deficiency, positive intrinsic factor antibodies, and strongly positive parietal cell antibodies supports a diagnosis of pernicious anemia, corresponding to APS type 3B. Positive gliadin antibodies along with duodenal biopsy findings of villous scalloping confirm active celiac disease, fulfilling criteria for APS type 3C. Additionally, a positive ANA test indicates systemic autoimmune activity, consistent with APS type 3D. 

Crucially, the patient’s adrenal function was preserved, with normal morning cortisol and an adequate Synacthen test response, effectively excluding APS types 1 and 2, both of which characteristically involve adrenal insufficiency [5]. The absence of mucocutaneous candidiasis and hypoparathyroidism further argues against APS type 1, a rare monogenic disorder predominantly presenting in childhood [2]. This clinical profile underscores the importance of screening for multiple autoimmune diseases once one endocrine autoimmunity is diagnosed, given the high risk of developing additional autoimmune conditions over time.

Unlike APS type 1, which is a monogenic autosomal recessive disorder resulting from AIRE gene mutations, APS type 3 is polygenic and more prevalent, especially among women in the second to fourth decades of life [2]. The coexistence of T1DM, autoimmune hypothyroidism, and celiac disease in this patient underscores the need for routine autoimmune screening in patients with established endocrine autoimmune conditions, even when overt symptoms are absent [6].

**Figure 6:**
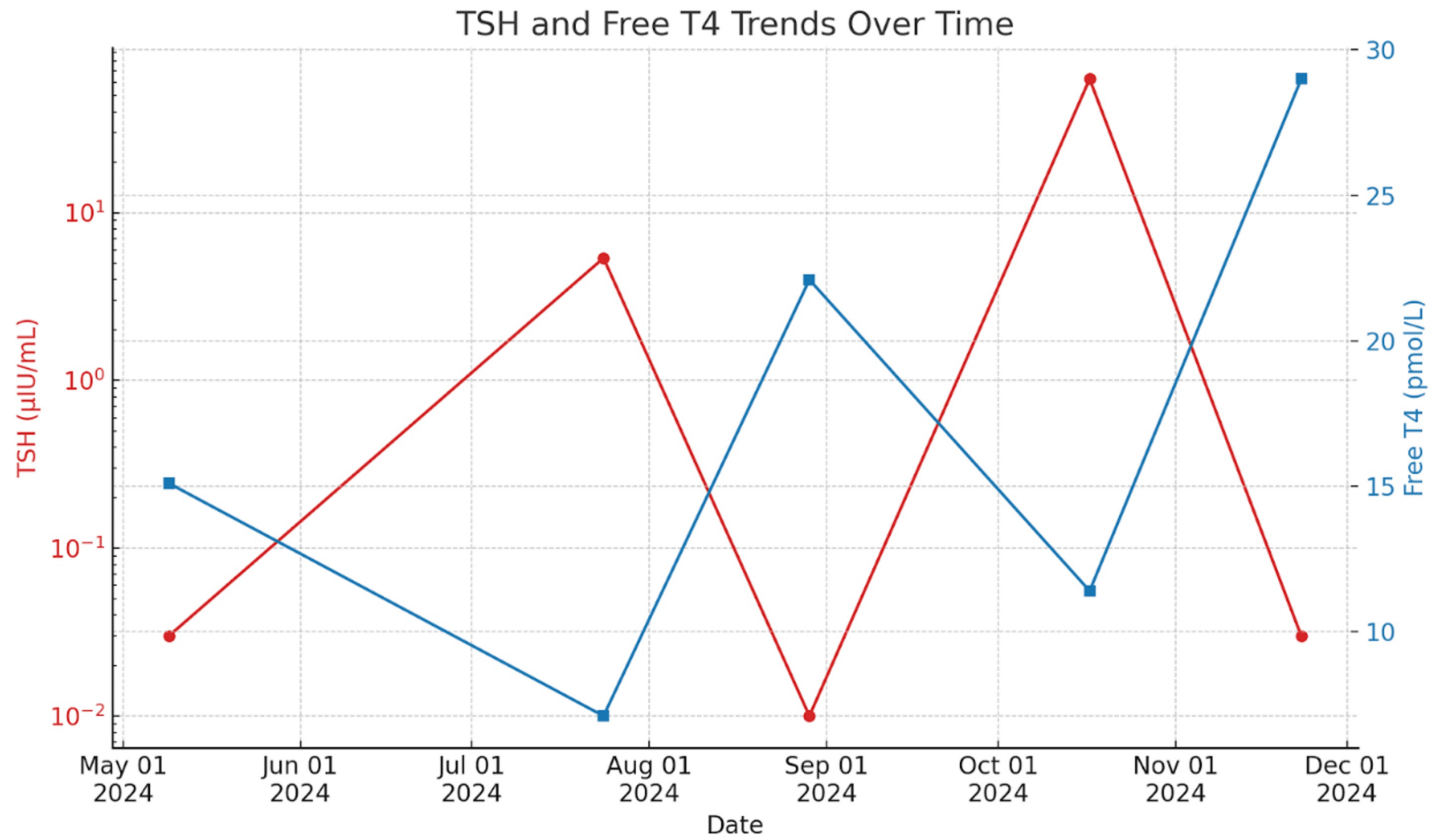
Graph showing the fluctuating levels of thyroid-stimulating hormone (TSH) and free thyroxine (free T4) over a defined period. The red line represents TSH values (mIU/L), while the blue line represents free T4 levels (pmol/L). The X-axis represents the timeline of testing, and the Y-axis denotes hormone concentration levels.

Fluctuating TSH and free T4 levels in a patient with APS type 3 may result from a combination of factors, including intermittent autoimmune thyroiditis activity, partial or evolving hypopituitarism, and inconsistent levothyroxine absorption, particularly in the presence of coexisting autoimmune gastrointestinal conditions such as celiac disease. Additionally, this pattern may reflect instability in thyroid hormone regulation due to autoimmune-mediated interference at multiple levels of the endocrine axis (Figure 6). Interestingly, these fluctuations may also hint at a deeper, less understood dysfunction within the hypothalamic-pituitary-thyroid axis itself, an underrecognized facet of autoimmune dysregulation that continues to challenge our current understanding [7].

Her elevated dose of levothyroxine may be attributed to multiple factors, including malabsorption secondary to untreated celiac disease, which reduces oral bioavailability of thyroid hormone [8]. Additionally, in subclinical hypopituitarism, the pituitary gland produces insufficient TSH, leading to secondary hypothyroidism. Since TSH is needed to stimulate the thyroid gland to produce thyroxine (T4), this deficiency results in lower endogenous thyroid hormone levels. As a result, patients may require higher doses of levothyroxine to compensate for the reduced stimulation and maintain adequate circulating thyroid hormone levels [9].

Non-adherence to a gluten-free diet not only exacerbates intestinal damage but can also contribute to poor glycemic control and nutritional deficiencies, worsening fatigue and increasing cardiovascular risk [10].

Moreover, her progression to proliferative diabetic retinopathy at a relatively young age illustrates the impact of suboptimal glycemic control and the additive burden of coexisting autoimmune conditions on long-term outcomes. While she has no clinical signs of adrenal insufficiency, periodic screening using ACTH stimulation testing is advisable, as adrenal failure can present insidiously but has life-threatening consequences [3].

## Conclusions

This case underscores the coexistence of multiple autoimmune conditions consistent with APS type 3, including components of subtypes 3A (diabetes), 3B (gastritis), 3C (celiac disease), and 3D (ANA positivity suggestive of systemic autoimmunity). While the patient’s presentation followed a relatively typical diagnostic course once autoimmune thyroiditis and diabetes were identified, it reinforces the importance of considering polyglandular syndromes in individuals with multiple autoimmune disorders. Although this is a single case, it highlights the value of comprehensive clinical assessment and interdisciplinary care in managing patients with overlapping autoimmune conditions. Early recognition may support proactive monitoring and tailored management, particularly in patients at risk for evolving adrenal or other gland involvement.
